# LOGIQA: a database dedicated to long-range genome interactions quality assessment

**DOI:** 10.1186/s12864-016-2642-1

**Published:** 2016-05-16

**Authors:** Marco-Antonio Mendoza-Parra, Matthias Blum, Valeriya Malysheva, Pierre-Etienne Cholley, Hinrich Gronemeyer

**Affiliations:** Equipe Labellisée Ligue Contre le Cancer, Illkirch, France; Department of Functional Genomics and Cancer, Institut de Génétique et de Biologie Moléculaire et Cellulaire (IGBMC), Illkirch, France; Centre National de la Recherche Scientifique UMR 7104, Illkirch, France; Institut National de la Santé et de la Recherche Médicale U964, Illkirch, France; University of Strasbourg, Illkirch, France

**Keywords:** HiC, Quality, Chromatin architecture

## Abstract

**Background:**

Proximity ligation-mediated methods are essential to study the impact of three-dimensional chromatin organization on gene programming. Albeit significant progress has been made in the development of computational tools that assess long-range chromatin interactions, next to nothing is known about the quality of the generated datasets.

**Method:**

We have developed LOGIQA (www.ngs-qc.org/logiqa), a database hosting quality scores for long-range genome interaction assays, accessible through a user-friendly web-based environment.

**Results:**

Currently, LOGIQA harbors QC scores for >900 datasets, which provides a global view of their relative quality and reveals the impact of genome size, coverage and other technical aspects. LOGIQA provides a user-friendly dataset query panel and a genome viewer to assess local genome-interaction maps at different resolution and quality-assessment conditions.

**Conclusions:**

LOGIQA is the first database hosting quality scores dedicated to long-range chromatin interaction assays, which in addition provides a platform for visualizing genome interactions made available by the scientific community.

**Electronic supplementary material:**

The online version of this article (doi:10.1186/s12864-016-2642-1) contains supplementary material, which is available to authorized users.

## Background

Today massive parallel DNA sequencing is used not only to decrypt the digital nature of genomes but, in combination with a variety of molecular biology techniques, it provides functional insights into a plethora of regulatory levels and functions, including epigenomics and protein-genome interactions (e.g., ChIP-seq, MeDIP-seq), global transcriptional activity (e.g., RNA-seq, GRO-seq, Ribo-seq), protein-RNA interactions (e.g., CLIP/RIP-seq), chromatin accessibility (e.g., DNase-seq, FAIRE-seq, ATAC-seq, MNase-seq) and the 3-dimensional chromatin organisation [HiC [1], ChIA-PET [2, 3]].

While data acquisition is not anymore an issue, today’s challenge is the availability of user-friendly computational solutions to interrogate and integrate - in a comparative manner - billions of data points from different types of functional genomics datasets. In fact, large consortia, like ENCODE, modENCODE, IHEC, NIH Epigenomics Roadmap provide enormous amounts of functional genomics data [4]. In addition, a great number of laboratories perform functional genomics studies in a diverse set of systems covering a large number of molecular targets, such that the number of genomics data linked to various cell/(patho)physiological functions increase exponentially in public repositories like the Gene Expression Omnibus (GEO [5]). However, despite the fact that these repositories contain huge amounts of functional genomics information their exploitation is seriously limited by (i) the lack of information on the quality of these datasets and (ii) the limited toolbox of exploratory computational resources.

In this context, we have developed previously a quality control system dedicated to ChIP-seq and enrichment-related datasets [6] (www.ngs-qc.org). Here we describe LOGIQA (www.ngs-qc.org/logiqa), a database hosting quality scores for long-range genome interaction assays accessible through a user-friendly web-based environment dedicated to quality-scored visualization of long-range interaction maps.

## Construction and content

### Principles used for quality assessment

**Fig. 1:**
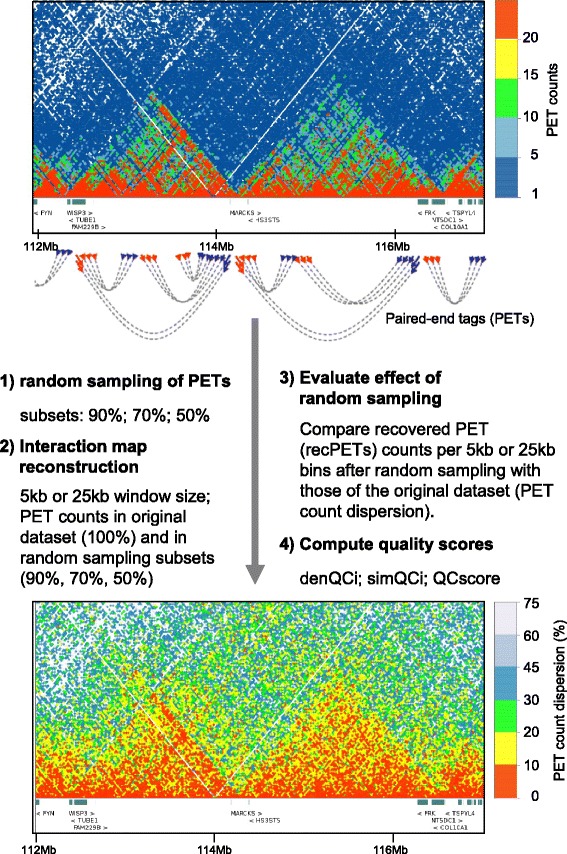
Principles in use for Quality Assessment. Total mapped paired-end tags (PETs) are first classified in intra-chromosomal and inter-chromosomal events. For quality assessment, only intra-chromosomal PETs spanning genome distances longer than 10 kb - referred here as filtered PETs - are considered. Random sub-sampling generates PET subsets corresponding to 90, 70 and 50 % of the original filtered PETs and the numbers of PETs in 5 kb or 25 kb size genomic windows is quantified. By comparing each of the PET counts/window in the various random subsets with that observed on the original dataset, the fraction of recovered PET counts (recPETs) after random sub-sampling and the dispersion from the theoretically expected values are calculated. Note that the expected values correspond to a decrease in the number of PET counts per window that is proportional to the random sub-sampling (e.g. recPETs/window =50 % when 50 % of filtered PETs are random sub-sampled). By evaluating the fraction of genomic windows with recPET count dispersions lower than a defined confidence interval (default value 10 %) global quality descriptors like the density and similarity quality indicators (denQCi, and simQCi respectively), as well as the global QCscore are computed. Overall these quality descriptors reflect the fractions of the observed long-range chromatin interactions (>10 kb), which are considered reproducible. On top of the panel: a chromatin interaction map derived from a HiC assay is depicted on the context of the observed PET counts (heatmap scale). On the bottom: After LOGIQA data treatment, the chromatin interaction map displays the inferred PET counts dispersion (in percent; heatmap scale). Notably, the bottom panel recapitulates the genomic contacts observed on the top panel, but in addition it provides a further information concerning their reproducibility over the multiple random sub-sampling assays accomplished during quality assessment

LOGIQA is based on the principles applied by the NGS-QC Generator to compute quality descriptors [6]; specifically this involves the assessment of multiple random samplings over long-range interaction readouts to infer numerical local and global quality scores (Fig. 1). In fact, the working hypothesis is that under ideal conditions, the reconstructed chromatin interaction maps from a subset of the mapped paired-end tags (PETs) should present the same patterns than those observed in the original map. Obviously, multiple factors can lead to a deviation from this optimal situation; one of them is the sequencing depth. Indeed, sequencing depths below a “saturation point”, as previously described for ChIP-sequencing assays[7], will lead to a decreased accuracy of chromatin interaction patterns. Importantly, applying this concept to long-range chromatin interaction assays provides a direct relationship between the sequencing depth and the confidence in predicting chromatin interactions. This confidence is herein referred to as the quality of the dataset under study.

Technically, we first selected unique PETs (excluding potential PCR-generated “clonal” reads), which participate in intra-chromosomal interactions longer than 10 kb. We thereby excluded PETs resulting from short-range chromatin interactions, which dominate chromatin interactomes (forming the diagonal in interaction maps) and would bias the quality assessment due to their over-representation. Indeed, Removal of PETs spanning >10 kb or >25 kb led to a direct correlation between the amounts of PETs per dataset and their associated QCscores (Additional file 1: Figure S1A). This correlated also with an improved visual quality and visibility of Topologically Associating Domains (TADs) in chromatin interaction maps (Additional file 1: Figure S1B). Next we established randomly sampled interaction PET subsets for defined fractions of the original population (90 %, 70 %, 50 %; described hereafter as s90, s70 or s50). After random sampling, intra-chromosomal interaction maps were reconstructed by assessing the number of PET counts within 5 kb or 25 kb bins. These two analytical windows enable quality assessment at two different resolutions and facilitate the comparison of different types of datasets; this concerns particularly HiC assays that are generated with different restriction enzymes or ChIA-PET assays involving sonication-sheared chromatin.

**Fig. 2:**
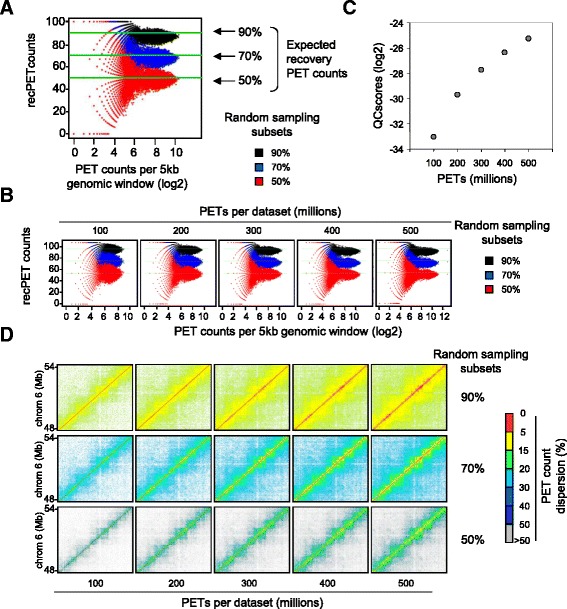
Assessing quality descriptors over long-range genome interaction assays. **a** Scatter-plot illustrating the fraction of PET counts recovered after random subsampling (Y-axis) relative to the original PET counts in 5 kb genome windows (X-axis). Note that genome windows with high PET counts contain PET levels close to the expected value; in contrast, the lower the PET counts, the higher is the deviation from this theoretically expected level. **b** Recovery scatter-plots assessed from datasets with increasing PET count levels (from 100 to 500 millions). Note that we generated these datasets by random sub-sampling of a large metafile (>600 million reads). **c** QCscores computed from datasets presenting increasing PET count levels (from 100 to 500 millions). The illustrated QCscores, computed from five independent replicates, present variation coefficients below 3 % (see Additional file 1: Figure S2). **d** Local displays illustrating chromatin interactions (chromosome 6, mm9) evaluated in the context of PET count dispersion levels (percentage) per genomic window (5 kb) relative to the expected recovery levels. Note that short-range genomic interactions (diagonal) show the lowest dispersion levels

Finally, global and local quality scores were computed by comparing the recovered PET counts per 5 kb or 25 kb bin after random sampling with those observed in the original dataset (Fig. 2a).

### Computing local and global quality indicators

Technically quality assessment is performed by first computing the recovered PET counts after random sampling as follows:

$$ recPETcounts=\left(\frac{samPETcounts}{oPETcounts}\right)*100 $$

where *samPETcounts* correspond to PET counts assessed after random sampling and *oPETcounts* correspond to those retrieved with the original dataset. Then it is used for computing the difference between the observed recovered PET counts after random sampling relative to that ideally expected (samd; which is equivalent to the random sampling density (90 %, 70 % or 50 %)):

$$ \partial PETcounts= samd- recPETcounts $$

The recovered PET count dispersion (δPETcounts) per genomic window is referred to as the local QC indicator, such that each evaluated genomic region (5 kb or 25 kb window) can be expressed by this quantitative readout assessed for a given random sampling subset analysis. Importantly, representing genome interaction maps in the context of PET count dispersions (*δPETcounts*) transforms the display into a uniform scale for comparing datasets generated at variable PET sequencing levels (e.g. PET count dispersion: 5-50 %).

Finally, while *δPETcounts* interaction maps provide a visual display of the quality associated to a given genomic region, they do not allow evaluation of the quality of the entire dataset. Therefore, we defined the following global quality descriptors:

### Density quality indicators (denQCi)

The fraction of genomic regions (5 kb or 25 kb window) in the random sampled datasets presenting *δPETcounts* lower than a defined threshold; which in the context of this study has been fixed at 10 %. Specifically, LOGIQA presents denQCi values computed for 90 %, 70 % and 50 % random samplings (denQC.90, denQC.70 and denQC.50 respectively).

### Similarity quality indicators (simQCi)

The ratio between two denQCis is used to evaluate their degree of similarity. Specifically, LOGIQA presents simQCi values computed for denQC.90 and denQC.70 relative to denQC.50 (simQC.90/50 and simQC.70/50 respectively).

Note that **denQCi** aims at quantifying the proportion of genomic regions that fluctuates in less than 10 % for a given random sampling. In fact, an s90 random sampling presents generally less variation from the original dataset, while the s50 subset will have the highest deviation. The **simQCi** measures the relative difference between denQC indicators computed at different random sub-sampling conditions. For instance, simQC.90/50 compares the denQC at 90 % to that computed at 50 % sub-sampling. In an ideal situation (saturation of the interactome readout), the fraction of genome interactions affected by the random sampling is identical at 90 % and 50 % and would yield a simQC = 1. While none of the evaluated datasets are at saturation, the closer this indicator is to 1, the lower is the difference of the denQC indicators between the two random sub-samplings and the higher is the dataset quality.

Intuitively, high quality datasets generally contain a high amount of genomics regions that are “robust” to the most severe 50 % random sub-sampling (i.e., they will display high denQC.50 levels); they will also show low differences between denQCis assessed at various random sub-sampling conditions (i.e., their simQC.90/50 and simQC.s70/50 will be close to 1). To integrate these two aspects on a single readout, we defined a global **QCscore**, which summarizes the previous metrics (denQCi and simQCi) into a single quality descriptor according to the following formula:

$$ QCscore=\left(\frac{denQC.50}{simQC.90/50}\right)\ast \left(\frac{denQC.50}{simQC.70/50}\right) $$

The **QCscore** provides a quality readout, in which the influence of both the denQC.50 and the simQCis computed for s90 relative to s50 (simQC.90/50), and s70 relative to s50 (simQC.70/s50) are represented.

### Quality scores computed for a variety of long-range chromatin interaction assays

Because of its universal principle, LOGIQA allows to compute quality scores for chromatin interaction datasets generated from a variety of techniques. Indeed, LOGIQA hosts currently QC scores for >250 publicly available HiC (including several variants of the original protocol, like in situ or capture HiC), but also several ChIA-PET (>50) and 4C-seq (>900) datasets.

## Utility

### Quality score validations

One of the principal motivations for the development of the present quality score system was to provide a numerical quality descriptor that can predict the optimal sequencing depth for long-range chromatin interaction assays. In fact, even though chromatin interaction assays are expected to require high sequencing depth [8, 9], to date there is no quantitative approach that can compare multiple HiC or similar assays in the context of their relative sequencing depths. The QCscores computed by LOGIQA solve this problem. To illustrate this point, we have constructed a HiC metafile composed of more than 600 million PETs and established subsets by random sampling (100, 200, 300, 400 and 500 million PETs), which were used for calibration of a quality scale. This calibration system reveals a direct negative correlation between sequencing depth and the deviation of the recovered PET count levels from the original dataset after random sampling (Fig. 2b; note the enlarged dispersions of the 100 million vs. the 500 million PET datasets) which translates into a gain of global QCscores for high PET counts (Fig. 2c). Importantly, the reproducibility of the computed global QCscores has been validated from multiple independent random samplings, for which the coefficient of variation was systematically <10 % (Additional file 1: Figure S2). This calibration revealed also the influence of the sequencing depth on PET count dispersion in a selected genome region, as illustrated for chromosome 6 in Fig. 2d, where the chromatin interaction maps reconstructed from different total PET counts are compared using a color-code for PET count dispersion.

**Fig. 3:**
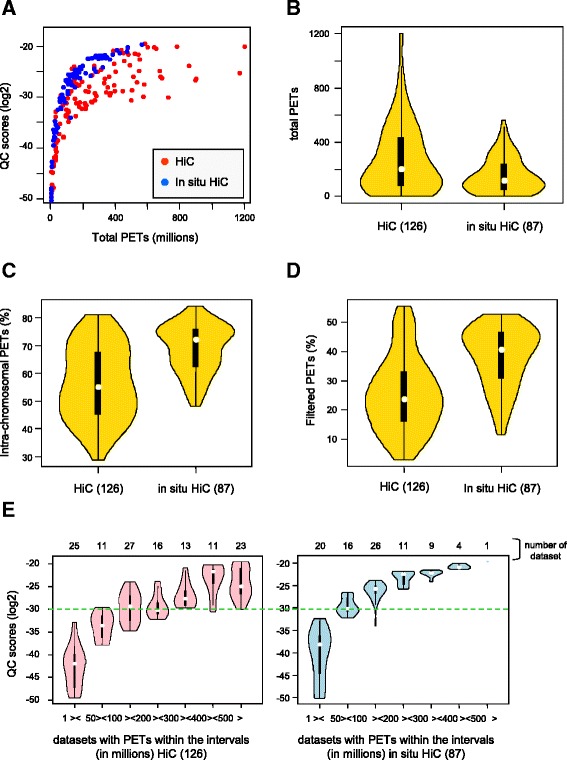
Quality scores assessed on 76 HiC and 71 in situ HiC assays evaluated in the context of total sequenced PETs. **a** Global Quality scores computed for HiC and in situ HiC assays relative to the total PETs. **b**, **c** and **d** Violin plots illustrating the number of total PETs (**b**), and the fraction of intra-chromosomal (**c**) and intra-chromosomal (**d**) filtered PETs. **e** Violin plots displaying the QC scores for HiC and in situ HiC datasets stratified for identical total PET intervals. The dashed horizontal green line demarcates the median QC score assessed for in situ assays with less than 200 million PETs

We next computed the quality scores for datasets that were reported to be of superior quality due to a modification of the technology, referred to as in situ HiC [10]. Specifically, these assays involve cell in situ proximity ligation, which reduces the frequency of random inter-molecular ligation. In this context, we compared QC scores computed for 126 HiC and 87 in situ HiC datasets in the context of their total sequenced PETs. The QC scores of the in situ HiC datasets were generally among the top for a given PET range (Fig. 3a, e) even though there was no clear separation in the quality of HiC and in situ HiC. Rather, it appears that the quality of HiC is more variable than that of in situ HiC, which were generally performed with lower total PETs (Fig. 3b). Our comparative analysis supported also the notion that there are less inter-chromosomal PETs in in situ HiC, as we observed on average more than 70 % intra-chromosomal PETs for in situ HiCs, while significantly less were seen in HiCs (Fig. 3c). Given that LOGIQA computes QC scores on the basis of intra-chromosomal PETs that span a genomic distance of above 10 kb (referred to as “filtered PETs”), we compared the two HiC technologies in the context of filtered PETs. We noted that in situ HiC assays generated on average significantly higher amounts of filtered PETs (~40 %) than HiC (~25 %) assays (Fig. 3d).

Albeit increasing the PET coverage can compensate for reduced QC scores, we were rather interested in comparing the QC scores of HiC and in situ HiC at comparable PET coverage (and thus similar sequencing costs). Notably, mean QC scores around −30 were attained by in situ HiC at a total PET coverage of 50 M to 100 M, while for HiC 100 M to 200 M PETs were required to reach this score (Fig. 3e; dashed green line).

**Fig. 4:**
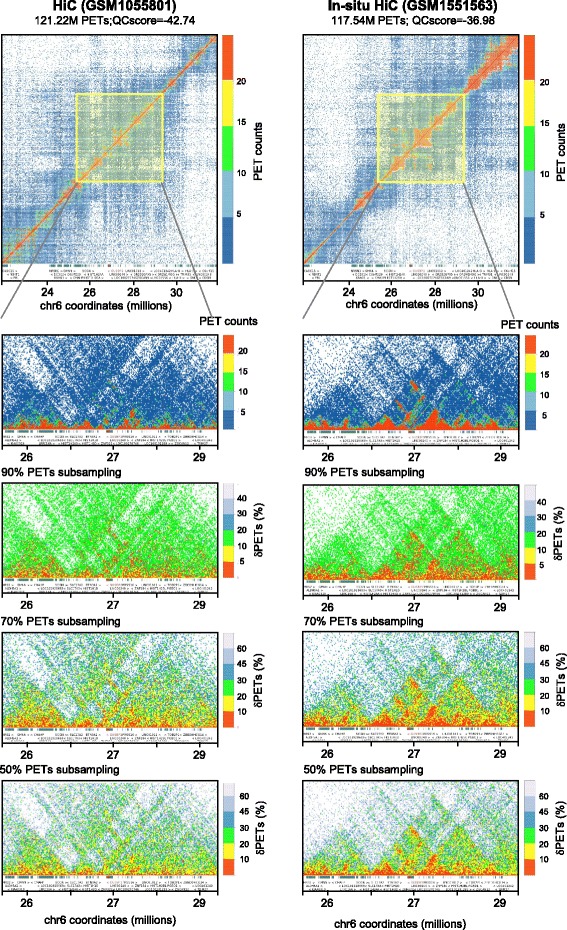
Chromosome 6 interaction maps displayed for two datasets presenting similar number of filtered PETs but different global QC scores. The illustrated HiC (GSM1055801) and in situ HiC (GSM1551563) datasets comprise about 120 million filtered PETs, nevertheless their global QC scores are different (higher quality for in situ than for the classical HiC assay). In both cases, large genome interaction views (top panels: 10 million bp), as well as closer views (5 million bp) clearly demonstrate the presence of more clearly defined topological domains in the in situ HiC dataset. Note that for the close-ups, both the PET count displays from the original datasets, as well as PET count dispersion displays (dPETs) of the random sub-samplings clearly illustrate the differences in quality of the interaction patterns

To demonstrate that the global QC score is a meaningful value also for local quality assessment we generated local genome interaction maps (chromosome 6, hg19) generated from two datasets with similar numbers of filtered PETs (~120 million) but significantly different global QC scores (Fig. 4). Importantly, the in situ HiC data formed clearly defined topological domains (TADs) for the illustrated region, which corresponds to the human histone gene cluster 1, while the dataset generated by classical HiC appeared less well defined. The visual perception of this difference is further enhanced when the graphic displays were generated from randomly sub-sampled fractions of the two original PET datasets. In fact, when 50 % of the PETs were used for reconstructing the chromatin interactomes, the TAD pattern was readily detectable by visual inspection in the in situ HiC assay for PET dispersion levels <10 %, while the classical HiC assay had PET dispersion levels >20 and a very blurred graphical presentation, in which no TADs could be identified.

Taken together, in situ HiC generates higher amounts of intra-chromosomal PETs and delivers at similar PET coverage better QC scores than HiC. Thus, the present comparative study with large populations of HiC datasets demonstrates the utility of the quality scores computed by LOGIQA.

### Quality scores as quantitative means for revealing heterogeneity among datasets

**Fig. 5:**
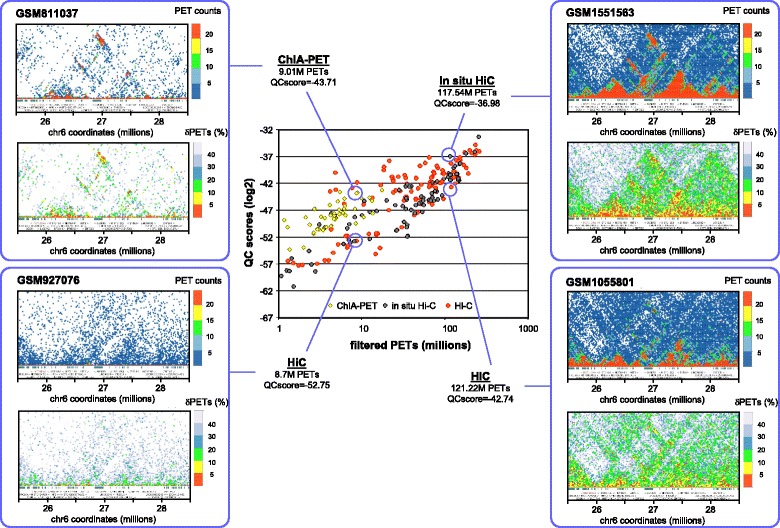
Comparison of a variety of long-range chromatin interaction datasets in the context of the sequenced paired-end tags (PETs). (*Center*) Scatter-plot illustrating the global quality scores for several long-range chromatin interaction assays in the context of the associated PET counts. (*Left and right panels*) To highlight the power of discrimination provided by global QC scores, the indicated datasets, chosen to represent low (left panels; ~9M PETs - GSM811037 & GSM927076) and high (*right panels*; ~120 PETs – GSM1551563 & GSM1055801) filtered PET count conditions, are illustrated in a local context (the panels show the histone gene cluster on chromosome 6). Local interaction maps generated by LOGIQA are depicted in PET counts (*top*) or PET count dispersion (bottom; %δPETs retrieved after 70 % random PET sub-sampling). Filtered PETs correspond to the number of intra-chromosomal contacts spanning a minimal genome distance of 10 kb

The LOGIQA database provides a global view of the relative quality of all long-range chromatin interaction assays, thus revealing the impact of the methodology, sequencing-depth and other technical/performance aspects that are specific to each individual assay. To illustrate the last point, we compiled the QC scores of multiple ChIA-PET, HiC and in situ HiC assays and displayed them relative to the filtered PETs used in the assays (Fig. 5, central panel). We then displayed contact maps for two pairs of datasets with largely distinct QC scores but similar filtered PET density - one pair comprised a ChIA-PET and a HiC (about 9 M filtered PETs) and the other an in situ HiC and a classical HiC (about 120 M filtered PETs). The illustrated maps correspond to the same region of chromosome 6 in which either the total PET counts or the PET count dispersions at 70 % sub-sampling are displayed (top and bottom panels, respectively, in each of the blue-framed boxes). It is very obvious from these displays that the in situ HiC GSM1551536 (top right) displays more confident chromatin interaction patterns than the HiC GSM1055801 (bottom right) and indeed, LOGIQA attributed a global QC score of −36.98 to the in situ HiC but only −42.74 to the HiC assay. Remarkably, the target-driven ChIA-PET GSM811037 presented a rather similar global QC score (−43.71) as HiC GSM1055801 even though a very low number of filtered PETs were obtained in this assay (~9 million) and TAD structures are clearly discernible in the connectivity maps (Top left), albeit with lower confidence than in the in situ HiC GSM1551536. In stark contrast to the ChIA-PET the connectivity map of HiC GSM927076 (Bottom left) that was generated with similar number of PETs does not reveal any TAD structures and received from LOGIQA the rather poor global QC score −52.75.

Overall, Fig. 5 clearly illustrates very convincingly the comparative power of the numerical QC scores computed by LOGIQA and their coherence with the visual impression obtained from chromatin contact maps.

While LOGIQA contains also quality indicators for more than 600 4C-seq assays, it is important to note that these values were computed differently. Since 4C-seq assays query all potential genomic interactions associated to a given genomic region - commonly referred to as “bait” - it resembles ChIP-seq assays, in which a target factor is used to define specific sites within the genome. Consequently, we performed quality assessments of 4C-seq similarly as for ChIP-seq assays using the NGS-QC Generator algorithm (for details see [6] or www.ngs-qc.org).

### LOGIQA provides a unique web access interface

**Fig. 6:**
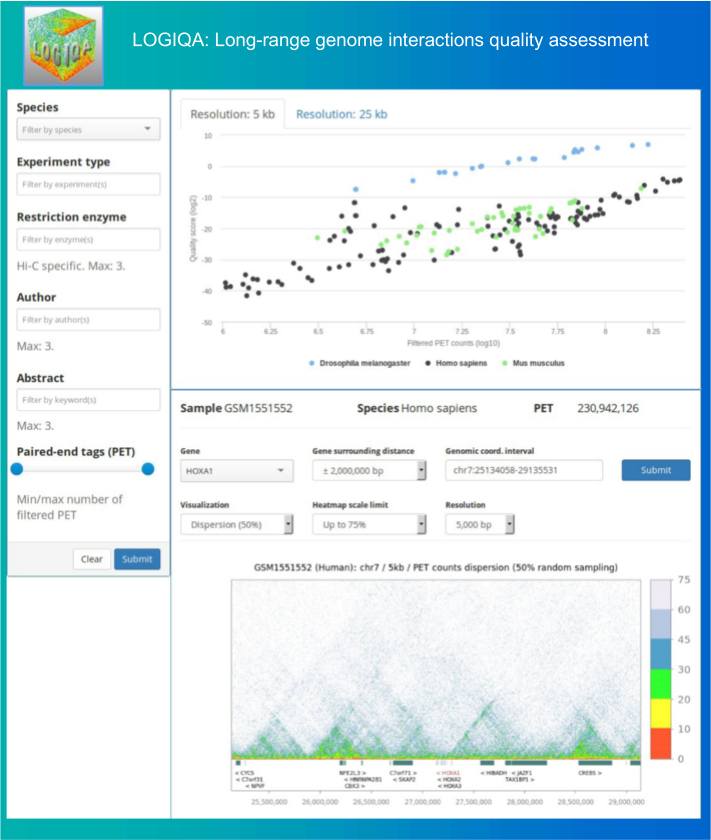
LOGIQA: A database hosting local and global quality scores assessed of long-range interaction assays. (*Top right*). Scatter-plot illustrating quality scores (y-axis, QC-score) for >160 HiC assays in the context of their associated paired-end tag (PET) counts (x-axis, log10). (*Top left*). Illustration of the LOGIQA query panel. (*Bottom panel*). Screenshot of the visualization tool displaying local chromatin interaction events depicted by their PET counts dispersion levels (heatmap scale)

In contrast to other computational solutions dedicated to visualize HiC or related datasets [10], users do not require to install any software to use LOGIQA. Furthermore, while a few other databases that host publicly available HiC and related assays became recently available [11, 12], LOGIQA is to our knowledge the first database of quality descriptors for a large collection of publicly available datasets. LOGIQA is a fully functional web-based system, which provides to users the quality scores for currently more than 900 publicly available datasets covering mouse, human and drosophila on a variety of long-range chromatin interaction assays. Specifically, global QC scores for all evaluated datasets are available in a scatter-plot format relative to their related PET counts, revealing the impact of genome size, sequencing-depth, and technical performance on the robustness and thus, quality of the data sets (Fig. 6 and Additional file 1: Figure S3).

To facilitate the retrieval of datasets, LOGIQA provides a user-friendly query panel covering items like species, type of experiment (e.g. in situ HiC), use of restriction enzyme for chromatin fragmentation, target molecule for ChIA-PET assays, name of (an) author(s), minimal/maximal PET counts to be retrieved, as well as a keyword search for the abstract of the corresponding publication(s).

Finally, LOGIQA provides a dedicated genome viewer, in which users can either select a defined gene (with user-defined upstream and downstream extensions), or provide genome coordinates (Fig. 6 and Additional file 1: Figure S4). The visualisation module displays either local QC dispersion readouts (for 70, 50 or 90 % random sampling conditions) or PET counts. The user can modify in both cases the associated heatmap scale and the genome window resolution (5 or 25 kb windows) (Additional file 1: Figure S5).

## Discussion and conclusions

Multiple features, which are at least in part interdependent, affect what can be considered as ‘quality’ of a long-range chromatin interaction assay. It is obvious that several experimental steps and procedures can be performed under more or less optimal conditions and that this will influence the final dataset. Some of the variables are purely experimental (crosslinking, restriction digest, end repair and biotin labelling in HiC; crosslinking, sonication and IP/antibody quality in ChIA-PET; generation of the sequencing library as well as sequencing coverage); others are bioinformatic (read alignment stringency). In this context, previous studies suggested that quality assessment in chromatin interaction assays could be performed by evaluating the alignment statistics, the frequency of dangling-end or self-circle PETs to reveal potential experimental problems during sample preparation, the levels of duplicated PETs as indicator of library complexity and PCR amplification bias, the fraction of intra over inter-chromosomal interactions and the frequency of long-range versus short-range intra-chromosomal interactions (see also [13]).

LOGIQA provides users with the possibility to retrieve the total PET counts, the fraction of unique PETs and number of intra and inter-chromosomal events. However, these are criteria that are more or less subjective, non-quantitative and non-cumulative; different users may value them differently. For example, while HiC assays may be judged subjectively as ‘good’ because they contain a high frequency of intra-chromosomal events, the variable ratio of long/short interaction PETs is generally not assessed. The quality assessment of LOGIQA fills this gap by computing the frequency of genomic contacts, which are in addition tested for “robustness” by random sub-sampling.

LOGIQA is based on the concept that we have previously presented for the assessment of quality scores for ChIP-seq and related assays [6]. The use of random sub-sampling of mapped PETs follows the same principle as for mapped reads from ChIP-seq assays. Specifically, this methodology is based on the concept of a “sequencing saturation point”, beyond which no new enrichments can be identified [7, 14]. This concept has been initially evaluated in a retrospective manner in ChIP-sequencing assays by assessing the number of significant binding sites retrieved when only a subset of the original sequenced reads is used for profile reconstruction (read random sub-sampling approach; [15]). In a similar manner we have shown empirically that in ChIP-sequencing assays genomic regions with high intensity levels followed a proportional decrease after mapped read sub-sampling [6].

LOGIQA is an independent tool that complements the NGS-QC database with quality score information associated to long-range chromatin interaction assays. In fact, the study of chromatin interactomes is rapidly gaining popularity in scientific community, as revealed by >170 publications indexed in Medline (November 2015) and >500 datasets deposited in GEO. While these numbers are small compared to several thousand ChIP-seq and related datasets, there is an obvious need of establishing quality standards for both types of datasets. Since our first release of the NGS-QC Generator tool in 2013, we have processed more than 30,000 public datasets and we expect to cover virtually all ChIP-seq datasets by 2016. Similarly, LOGIQA will be expanded to cover all available HiC datasets and other type of datasets, like ChIA-PET. Ultimately, we will provide to users a cross-visualisation platform that displays datasets processed by the NGS-QC Generator together with those retrieved by LOGIQA such that users can explore long-range chromatin interaction maps in the context of available ChIP-seq and related datasets. Together, LOGIQA and NGS-QC Generator represent powerful tools for quality-guided exploration of public repositories dedicated to functional genomics datasets.

## Availability and requirements

### Database availability

LOGIQA is available trough a dedicated web access : www.ngs-qc.org/logiqa.

### Ethics approval and consent to participate

Not applicable

### Consent for publication

Not applicable

## Additional file

Additional file 1: Figure S1. Influence of the short-range PET distance on the assessment of LOGIQA QCscores. **Figure S2.** Global QC scores reproducibility evaluated over multiple PETs’ random sub-sampling. **Figure S3.** Global overview of the LOGIQA web application. **Figure S4.** Visualization panel (Interaction map). **Figure S5.** Genome interaction maps for the dataset GSM1551643 assessed at 5kb and 25kb bins resolution. (PDF 1952 kb)

## Abbreviations

ChIP-seq: chromatin immunoprecitipation combined with massive parallel sequencing
LOGIQA: Long-range Genome Interactions Quality Assessment
PETs: paired-end tags
