# Supplementary material for: LOGIQA: a database dedicated to long-range genome interactions quality assessment
Source: BMC Genomics. 2016 May 16;17:355. doi: 10.1186/s12864-016-2642-1 (PMC4868109; doi:10.1186/s12864-016-2642-1)
Supplement: Additional file 1: Figure S1. — Influence of the short-range PET distance on the assessment of LOGIQA QCscores. Figure S2. Global QC scores reproducibility evaluated over multiple PETs’ random sub-sampling. Figure S3. Global overview of the LOGIQA web application. Figure S4. Visualization panel (Interaction map). Figure S5. Genome interaction maps for the dataset GSM1551643 assessed at 5kb and 25kb bins resolution. (PDF 1952 kb) [file 12864_2016_2642_MOESM1_ESM.pdf]

LOGIQA: A database dedicated to Long-range Genome Interactions Quality Assessment

Marco-Antonio Mendoza-Parra*, Matthias Blum, Valeriya Malysheva, Pierre-etienne Cholley and Hinrich Gronemeyer*

Equipe Labellisée Ligue Contre le Cancer, Department of Functional Genomics and Cancer, Institut de Génétique et de Biologie Moléculaire et Cellulaire (IGBMC), Centre National de la Recherche Scientifique UMR 7104, Institut National de la Santé et de la Recherche Médicale U964, University of Strasbourg, Illkirch, France.

*Corresponding authors:
Marco Antonio Mendoza-Parra e-mail: marco@igbmc.fr
Hinrich Gronemeyer e-mail: hg@igbmc.u-strasbg.fr

Supplementary material

Figure S1. **Influence of the short-range PET distance on the assessment of LOGIQA QCscores.**

Figure S2. Global QC scores reproducibility evaluated over multiple PETs' random sub-sampling.

Figure S3. Global overview of the LOGIQA web application.

Figure S4. Visualization panel (Interaction map).

Figure S5. Genome interaction maps for the dataset GSM1551643 assessed at 5kb and 25kb bins resolution.

A

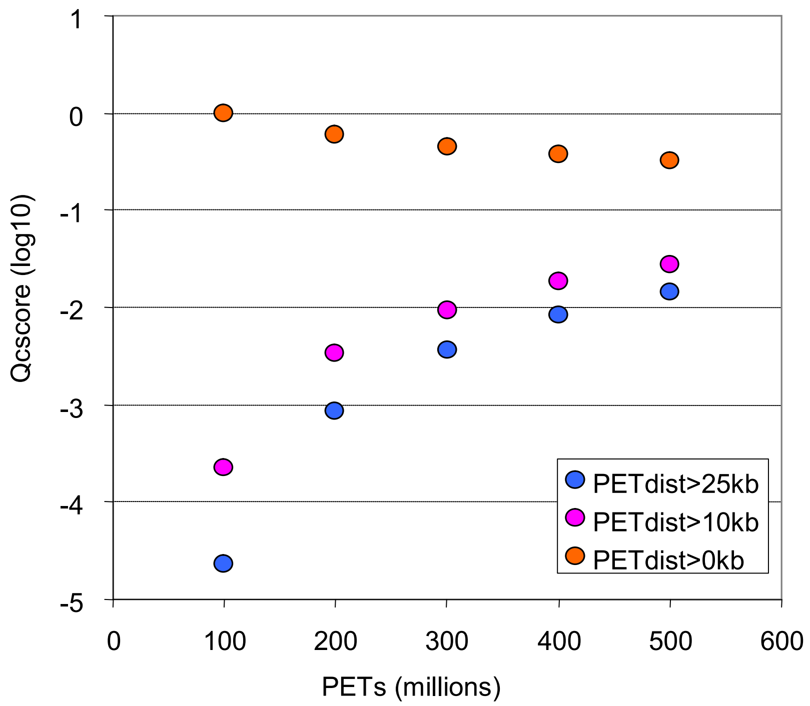

B

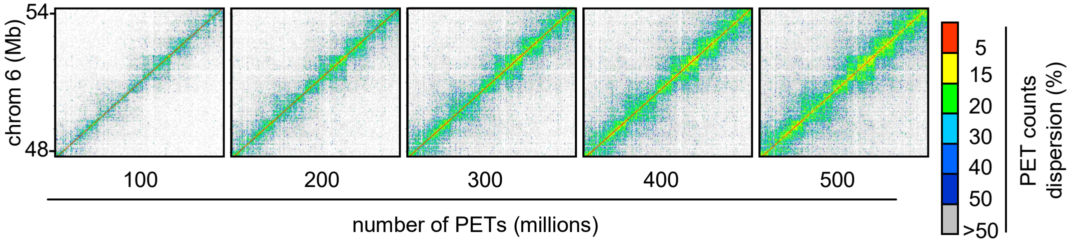

Figure S1. **Influence of short-range PETs on the assessment of LOGIQA QCscores.**
(A) QCscores were computed for 5 datasets comprising increasing amounts of PETs (from 100 ot 500 million) using three different conditions; (i) PET distance (PETdist) >25kb (blue circles); (ii) PETdist>10kb (pink circles)and (iii) including all PETs (PETdist>0kb; orange circles). Note that only in case (i) and (ii) the QCscores increase proportionally to the number of PETs per dataset. **(B)** Chromatin interaction maps (chromosome 6) illustrating the gain in confidence (low PET count dispersion) as a consequence of the increase of the number of PETs per assay. Note that in all cases, the PETs spanning the shortest distances (defining the diagonal of the display) show the lowest PET count dispersion.

A

PETs	Qcscore.5	stdev	CV(%)	Qcscore.10	stdev	CV(%)	Qcscore.15	stdev	CV(%)
100000000	8.167E-13	4.24E-14	5.19	1.13E-10	4.84E-12	4.28	5.26E-12	1.55E-13	2.94
200000000	6.998E-12	4.38E-13	6.26	1.15E-09	2.25E-11	1.96	1.50E-10	1.74E-12	1.16
300000000	2.163E-11	7.93E-13	3.67	4.43E-09	6.11E-11	1.38	1.34E-09	9.47E-12	0.71
400000000	4.456E-11	1.50E-12	3.36	1.16E-08	2.21E-10	1.90	6.45E-09	3.48E-11	0.54
500000000	7.507E-11	3.02E-12	4.02	2.47E-08	3.23E-10	1.31	2.13E-08	3.56E-11	0.17

B

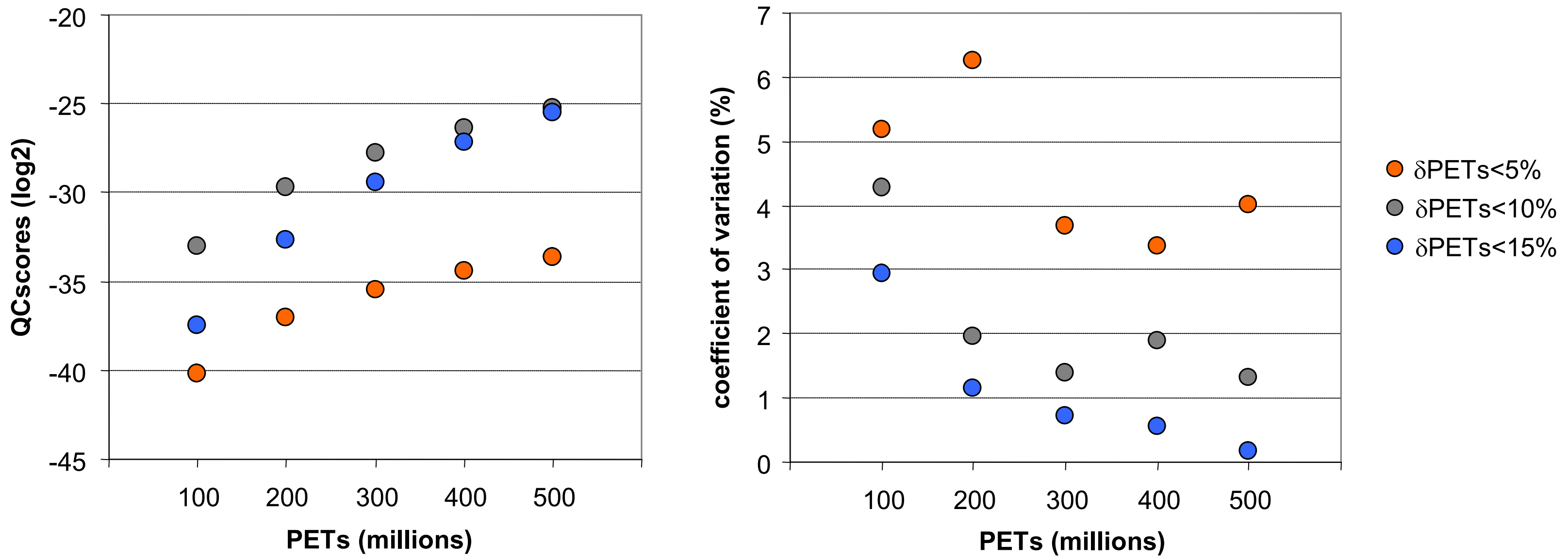

Figure S2. Global QCscores reproducibility evaluated for multiple random sub-samplings of PETs.
A Hi-C metafile composed of more than 600 million PETs was used to generate five randomly sampled subsets (100 to 500 million PETs respectively). For each of them, QC scores were computed for five independent replicates, then their average, standard deviation as well as the coefficient of variation (CV in percent) were assessed as illustrated in panel A. Scatter-plots in panel B illustrate the direct correlation between the total PETs and the inferred QC scores (left panel), as well as their low coefficient of variation among replicates (right panel). These analyses were computed at three different PET count dispersion levels (5, 10 and 15%).

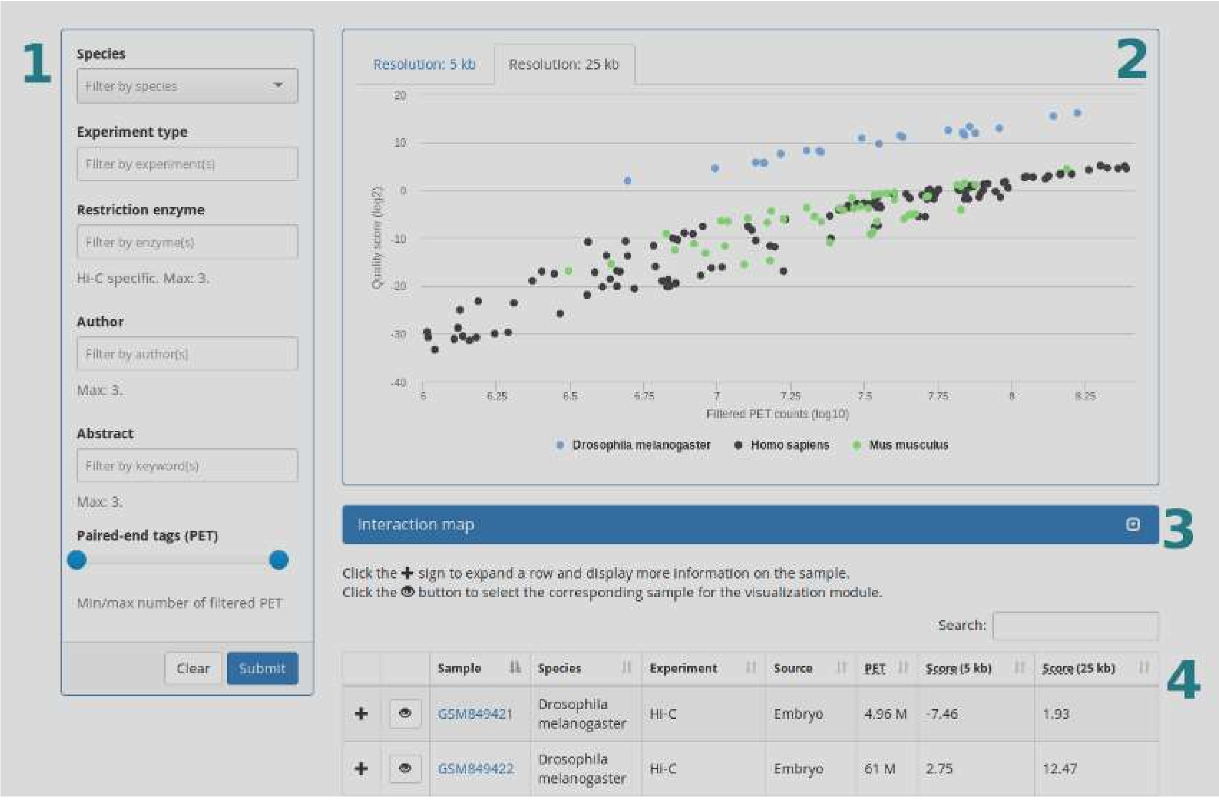

Figure S3. Global overview of the LOGIQA web application. The elements composing the main view in LOGIQA are listed as follows:

1) Filter panel: search experiments by using specific filters (e.g. restriction enzyme, authors, or minimum/maximum number of PET).
2) Global quality scatter-plot: display the quality scores computed per assay relative to their associated PET counts. This global information is available for 5 kb and 25 kb genome window resolutions. Each point, representing a single experiment, can be clicked to select the experiment for the visualization module.
3) Visualization panel (Interaction map): expand/collapse by clicking the blue header; visualize interaction maps at different resolutions and user-defined genomic regions.
4) Experiments table: display information associated with the datasets displayed in the scatter-plot. Users can access to further information by clicking on the "+" sign.

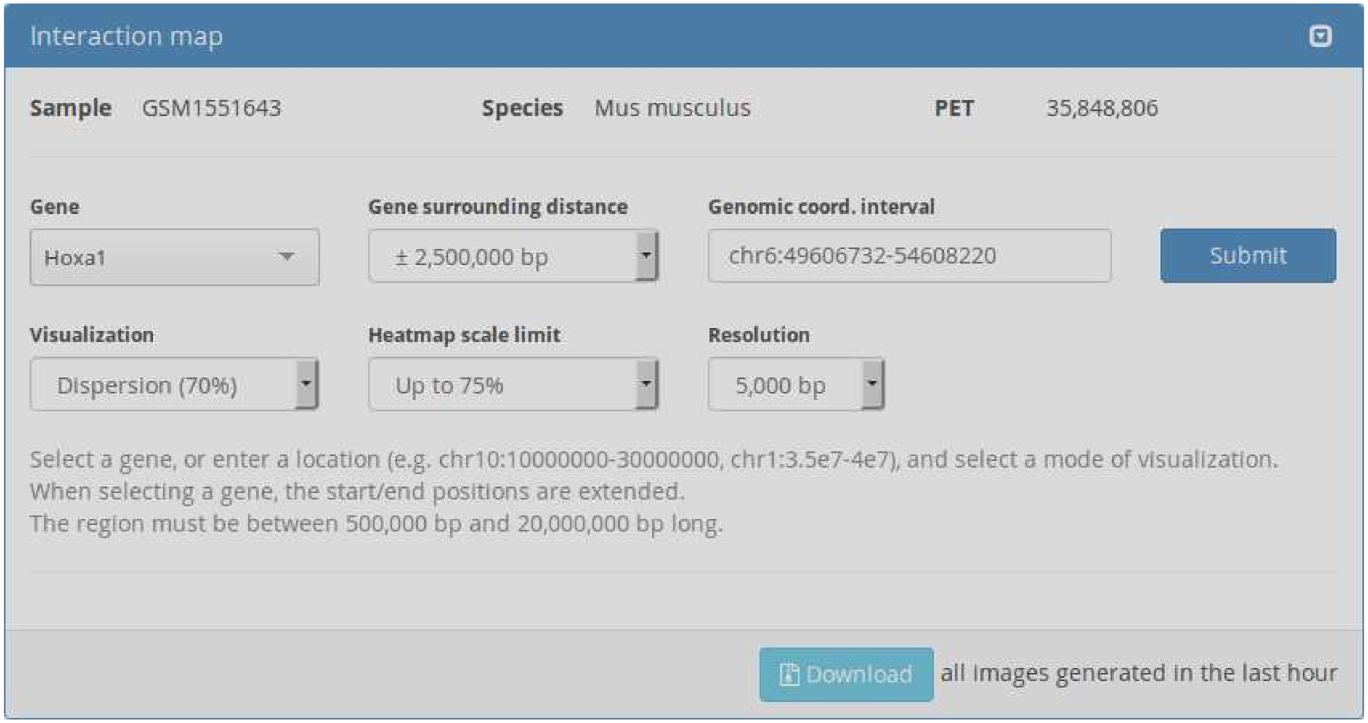

Figure S4. Visualization panel (Interaction map): LOGIQA provides a user-friendly web-access for local chromatin interaction views. Users select an experiment from either the global QCscores scatter-plot or from the experiments table. The local view is defined for a user-selected gene or defined genomic coordinates. Users may select the visualization style (PET counts dispersion or PET counts), the heatmap scale limit, as well as the resolution.

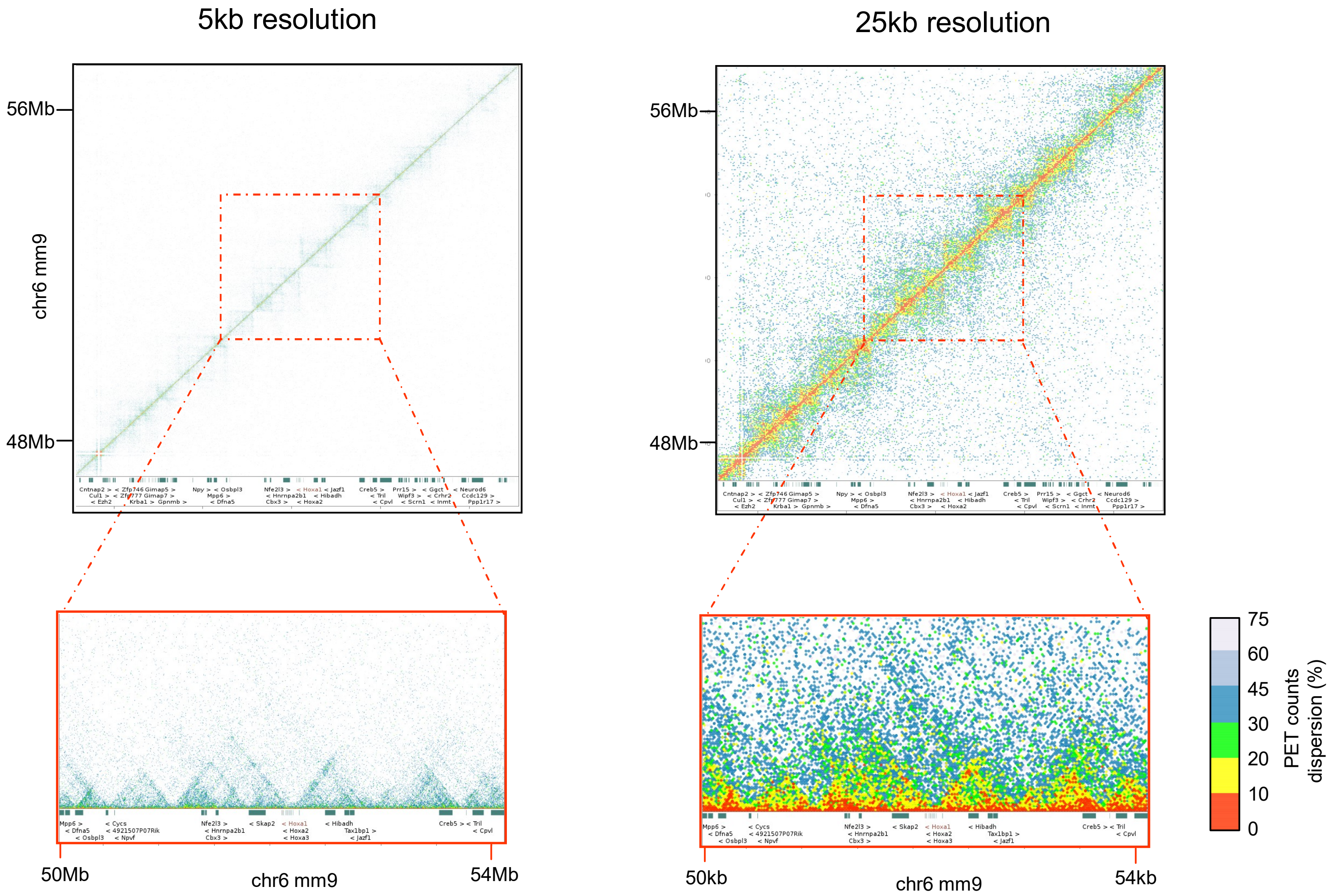

Figure S5. Genome interaction maps for the dataset GSM1551643 assessed at 5kb and 25kb bins resolution. The heatmap illustrates the local QC confidence per assessed bin (PET counts dispersion when comparing the 70% random sampling with the original PET counts).
